# The development of a model of training in child psychiatry for non-physician clinicians in Ethiopia

**DOI:** 10.1186/1753-2000-8-6

**Published:** 2014-02-25

**Authors:** Markos Tesfaye, Mubarek Abera, Christine Gruber-Frank, Reiner Frank

**Affiliations:** 1 Department of Psychiatry, College of Public Health and Medical Sciences, Jimma University, Jimma, Ethiopia; 2 Global Mental Health Group, Centre for International Health, Ludwig Maximilians University, Munich, Germany

**Keywords:** Child mental health curriculum, Low income country, Ethiopia, Teaching, Capacity building, WHO mhGAP intervention guide, Program evaluation

## Abstract

**Background:**

The lack of trained mental health professionals has been an important barrier to establishing mental health services in low income countries. The purpose of this paper is to describe the development and implementation of child psychiatry training within a graduate program in mental health for non-physician clinicians in Ethiopia.

**Methods:**

The existing needs for competent practitioners in child psychiatry were identified through discussions with psychiatrists working in Ethiopia as well as with relevant departments within the Federal Ministry of Health Ethiopia (FMOHE). As part of a curriculum for a two year Master of Science (MSC) in Mental Health program for non-physician clinicians, child psychiatry training was designed and implemented by Jimma University with the involvement of experts from Addis Ababa University (AAU), Ethiopia, and Ludwig-Maximillian’s University, (LMU), Germany. Graduates gave feedback after completing the course. The World Health Organization’s (WHO) Mental Health Gap Action Program (mhGAP) intervention guide (IG) adapted for Ethiopian context was used as the main training material.

**Results:**

A two-week child psychiatry course and a four week child psychiatry clinical internship were successfully implemented during the first and the second years of the MSC program respectively. During the two week psychiatry course, trainees learned to observe the behavior and to assess the mental status of children at different ages who had a variety of mental health conditions. Assessment of the trainees’ clinical skills was done by the instructors at the end of the child psychiatry course as well as during the subsequent four week clinical internship. The trainees generally rated the course to be ‘very good’ to ‘excellent’. Many of the graduates have become faculty at the various universities in Ethiopia.

**Conclusion:**

Child psychiatry training for non-physician mental health specialist trainees was developed and successfully implemented through collaboration with other universities. The model of institutional collaboration in training mental health professionals in the context of limited resources provides a useful guide for other low income countries where there is scarcity of psychiatrists.

## Background

The Lancet series on Global Mental Health 2007 and 2011 highlighted human resource issues as the most crucial factors for the expansion of mental health services in developing countries [1,2]. To develop effective mental health programs for children and adolescents in a given population, an integrated, multi-tier approach with an emphasis on primary care has been suggested [3]. Implementing such programs successfully requires the availability of trained professionals as well as a political commitment at the national level [4]. However, the lack of government policy, inadequate funding and scarcity of trained professionals in the field of child and adolescent mental health still continue to be challenges in low income countries [1].

Ethiopia is a sub-Saharan African country with an estimated population of nearly 94 million [5]. It is one of the world’s poorest countries with per capita income of 370 USD [6]. Mental health care is one of the most underserved areas of health services. Currently, there are approximately 46 psychiatrists practicing in the country, 461 psychiatric nurses, 14 psychologists, 3 clinical social workers, and no occupational therapists [7]. Over 85% of the Ethiopian population live in rural areas and have limited access to any mental health services. However, the majority of specialist mental health care services including child and adolescent psychiatric services are based in the capital, Addis Ababa [7].

In response to the limited resources for mental health in low income countries, the WHO’s mhGAP intervention guide (IG) has been developed and published for implementation of services at primary care level by non-specialists [8]. The intervention guide has been developed with the assumption that district health workers would feel more comfortable providing this type of care when they learn that interventions are simple and applicable to their context; and therefore, can be integrated within the existing health care system [8]. However, successful implementation of mental health care at the primary care level requires specialists for the training and ongoing supervision of primary care workers [8,9]. Ethiopia’s Federal Ministry of Health (FMOHE) incorporated mhGAP as the foundation for psychiatric services at the primary care level within the national mental health strategy [7]. In preparation for national implementation, the FMOHE, in collaboration with the WHO has adapted the mhGAP-IG [7,8] to the local context of the health care system. Similar to other low income settings, there has not been an adequate number of psychiatrists within Ethiopia’s mental health care system to provide training for other types of providers [10].

Fricchione et al. [11] have suggested that the optimal approach to building capacity in global mental health care requires partnerships between professional resources in high-income countries and promising health-related institutions in low- and middle-income countries [11]. For example, the successful establishment of a psychiatry residency program at Addis Ababa University (AAU) with support from the University of Toronto (TAAPP) provides a model for training mental health specialists within the context of a developing country [12].

Some low in-come countries have attempted to train practicing pediatricians through workshops to address the issue of human resource for child and adolescent psychiatry [13]. However, there is still a lack of documentation on the implementation of pre-service training in child and adolescent psychiatry by incorporating it into postgraduate training curriculum in low in-come settings [3].

The four-tier Ethiopian health system, with primary care centres being the lowest level of care with district (primary) hospitals, regional hospitals, and referral hospitals being subsequent increasing levels of care, has a critical shortage of professionals. The few psychiatrists graduating from AAU have been responsible for setting up mental health services at the regional and referral hospitals. However, the district hospitals which are rapidly increasing in number throughout the country need mid-level mental health specialists. In addition, several new university programs training medical nurses and health officers (non-physician clinicians) lacked trainers for psychiatry courses for these undergraduate students. Health officers and nurses who hold bachelor’s degrees and who are trained to provide primary health care are considered to be ideal candidates to be trained in mental health to address the identified gaps [14].

A model similar to TAAPP was implemented in January 2010 by Jimma University (Ethiopia) in cooperation with Amanuel Mental Specialized Hospital, Addis Ababa University (Addis Ababa, Ethiopia), Ludwig-Maximilians-University (Munich, Germany), and Brigham and Women’s Hospital (Boston, MA, USA) aimed at training non-physician mental health specialists. This program, Master of Science in Integrated Clinical and Community Mental Health, attempts to produce mid-level professionals with competencies in both adult and child psychiatry. The lack of child psychiatric training for primary care health workers makes it imperative to describe the structure and content of this program so as it can be incorporated into the curricula of similar programs.

### Setting

The Jimma zone (sub region), located in the south-western part of Ethiopia, is one of the zones in the Oromiya National Regional State. According to the 2007 census, the total population of the zone is over 2.4 million people [15]. Jimma University, one of the state run public universities in the country, has a Medical School with a Department of Psychiatry. The latter has been responsible for running a recently upgraded psychiatric facility within the auspices of Jimma University Specialized Hospital (JUSH).

JUSH, located in Jimma City, and is the only place where residents of Jimma zone can get modern psychiatric service. The psychiatric facility within JUSH has approximately 30 inpatient beds, and provides outpatient services for children, adolescents and adults. In 2008, the staff consisted of 1 psychiatrist, 2 general practitioners and 3 psychiatric nurses.

The medical school at Jimma University had an established collaboration with LMU which aimed at improving undergraduate medical education*,* curriculum development for graduate education, graduate training and faculty training since 2002 [16]. By 2009, LMU launched the Center for International Health (CIH) with a broader goal of strengthening the existing international academic collaborations. Consequently, supporting the graduate program in mental health at Jimma University gained a special focus.

### Purpose of the paper

The purpose of this paper is to describe the development and implementation of child psychiatry training within the graduate program described above at Jimma University, Ethiopia.

## Methods

### Development of the graduate program

The need to establish graduate training in mental health for mid-level health workers was reaffirmed by the FMOHE at a consultative meeting organized by Jimma University and other stakeholders in June 2008. The candidates who were recruited for enrollment into this program were required to have completed a degree in nursing or health officer training. After completion of the two-year training, graduates were expected to establish and to run services within district hospitals and also to provide training and supervision to primary care providers working within the catchment area of their hospital. They were also expected to conduct mental health related research and to be administrators at different levels of the regional health bureaus.

The Department of Psychiatry at Jimma University developed a draft curriculum for the planned graduate program with the involvement of local and international collaborators [17]. Specific courses were developed with the involvement of experts in the respective fields see the Master of Science (MSC) in integrated clinical and community mental health course organization Section.

### Master of Science (MSC) in integrated clinical and community mental health course organization

Applied Neuroscience

Psychopharmacology

Social Work and Family Assessment

Normal Psychology and Psychological Development

Clinical Psychiatry I, II, III and IV

Ethics, Law and Professionalism in Psychiatry

General Adult Psychiatry

Social Determinants of Health

Child and Adolescent Psychiatry Course

Counselling Psychology

Research Methods in Mental Health

Mental Health Services Management

Special Topics in Psychiatry

Consultation-Liaison Psychiatry

Principles of Psychotherapy

Clinical Child and Adolescent Psychiatry

Clinical Addiction Psychiatry

### Child psychiatry training

Initially there was no child psychiatrist faculty at Jimma University. Therefore, the child psychiatry part of the MSC program was developed in consultation with experts from AAU as well as LMU. A general consensus was reached on a two credit hour teaching (40 hours over two-weeks) child psychiatry course during the first year of the MSC training followed by a four-week child psychiatry clinical internship during the second year of the training. Course objectives and a course description were developed. Methods of teaching, reference textbooks and methods of assessment of students were also defined and documented within the MSC curriculum.

Overall objectives of the child psychiatry training

1. To describe the epidemiology and etiology of psychiatric disorders commonly occurring during childhood and adolescence,

2. To recognize the clinical features of psychiatric disorders commonly occurring during childhood and adolescence,

3. To demonstrate the ability to manage psychiatric disorders commonly occurring during childhood and adolescence

### Child psychiatry course description (First year)

The first year course in child and adolescent psychiatry aimed to teach the students to be familiar with mental and behavioral disorders in children and adolescents as well as the skills needed to assess psychiatric disorders in this age group [14]. The course emphasized pervasive developmental disorders, intellectual disability, childhood behavioral and emotional disorders, enuresis, attention deficit hyperactivity disorder, and child abuse and neglect. Adolescent medicine issues such as sexual development, drug use, and risk factors and prevention of suicide also were a focus [14].

### Curricular materials

The textbook of the International Association of Child and Adolescent Psychiatry and Allied Professions (IACAPAP), which takes [18] into account cultural differences and systems of care in countries with low resources, is available on the internet without cost. This CAP textbook was used as a primary reference.

The WHO mhGAP intervention guide on priority conditions in mental health also was utilized as a core reference on content [8]*. Principles of general care* that apply to all mental health problems are outlined at the beginning of the IG. Algorithms are provided for assessment, decision making and management. Two sections deal with mental health in children and adolescents: one on developmental delay and pervasive developmental disorders; and another on behavioral problems. The section on epilepsy also applies to children and adolescents [8]. During the clinical experience, materials such as pencil and paper, small blocks, toys, balls, etc. were needed as well as a video camera.

### Methods of teaching and assessment

For the child psychiatry course didactic lectures, case discussions and seminar presentations were designed [11]. A didactic approach had been used in an elective course for German medical students by a visiting child psychiatrist [19]. The latter was used as a model for the classroom didactic teaching. In addition, teaching clinical skills through video examples and demonstrations with child patients in the psychiatric clinic were incorporated [20]. Trainees were assessed by observation of their clinical work as well as their seminar presentation skills. For the child psychiatry second year clinical experience, a clinical internship was designed where the trainees under supervision had to assess and manage children coming to the psychiatric outpatient clinic.

### Overall course/program/evaluation

The overall outcome measures included trainees’ satisfaction with the program, and the expansion of child mental health training and services throughout the country. Therefore, the evaluations included direct feedback from the trainees immediately after they had taken the course and later, when they were practicing at their respective places of work after graduation, as well as the successful establishment of the capacity to run the child psychiatry training by Jimma University’s local faculty.

### Resources and funding

The MSC program curriculum was approved by the Jimma University Senate before it was implemented in January 2010. Signing formal agreement with collaborators to ensure commitment was an essential precondition for the program to be approved by the Senate. The program is funded by the Federal Ministry of Education, Ethiopia through Jimma University. Trainees were obliged to serve in a public institution after completion of their training; therefore, they were not required to pay any tuition fees. For child psychiatry training, the expenses of visiting professors were covered by Jimma University and CIH-LMU. Also, Jimma University provided perdiems for trainees when they had to travel for training outside of Jimma City.

### Implementation

#### Child psychiatry course

Before taking child psychiatry course the students have worked in adult inpatient and outpatient services for approximately 10 months under the supervision of a faculty psychiatrist (MT). English is used for teaching and medical records. The first child psychiatry course was taught by visiting child psychiatrist (RF) and children’s nurse (CGF) both from Germany (supported by CIH-LMU) who came to Jimma University for two weeks in November 2010. During the subsequent courses in February 2012 and 2013, MA (one of the trainees of the course offered in 2010) was the co-teacher to RF.

The course began with introductory didactic lectures on normal child development and on the broad categories of psychiatric disorders in childhood. The lecture material was given to the trainees as a hardcopy including a list of electronically available references. Students were assigned topics on specific childhood mental health problems which they had to prepare and present on during the seminars to their classmates later in the course.

The main emphasis of the child psychiatry course was to teach skills on how to: assess the emotions, behavior and functioning of children; develop management plans for specific mental health conditions, and engage and work with patients, families and other providers based on the WHO mhGAP-IG guidelines [8]. The first week of the course was devoted to developmental delay in children and general assessment techniques and approaches. The second week to behavioral disorders and developing management plans. See Child psychiatry course agenda section.

#### Child psychiatry course agenda

***Developmental Delay****-week 1*

How to assess cognitive abilities in adult patients

Treatment/rehabilitation in Intellectual Disability - basic concepts

Prevention of Intellectual Disability - one example

Epilepsy clinical picture*

Epilepsy – treatment options*

***Behavioral Disorders****– week 2*

Prevalence of behavioral disorders in children in Ethiopia

Interventions for emotional disorders: universal**

Interventions for behavioral disorders: universal**

Child Abuse

Disturbing behavior - clinical picture**

Disturbing behavior – intervention**

* Section epilepsy [8].

**See Kieling et al. [1].

The teaching method was primarily interactive with active involvement of trainees using the following methods:

1. Videotapes: these were prepared by RF and CGF in clinical settings. After parental consent and child assent, children attending the psychiatric outpatient clinic were recorded while doing activities such as drawing or painting, playing with a small ball, folding papers, or completing puzzles. Also, the videotapes included demonstrations of child psychiatric interviews and neurological examinations.

Trainees learned to observe and describe the behavior of a child in a structured manner. Patients at different ages with a variety of disorders were demonstrated in short video examples. Videos of patients recorded in the psychiatric facility were shown in the class room setting. Students were asked to write up the mental status exam on the patient. One student from the group presented his/her description, which was then written on the blackboard as a prompt for group discussion and constructive feedback. Discrepant perspectives were clarified by reviewing the video again and further discussion. Discrepancies in observation usually represented different aspects of the same patient. Supportive interventions that would be appropriate for the child patient by the family and professionals were discussed based on the information from the video-sequences.

2. Clinical interviews: trainees observed interviews of children and their parents in the clinic setting by the faculty. A children’s nurse (CGF) helped to engage the children with play activities. Building blocks, toy cars and bottle caps were attractive for younger children and for children with severe developmental delays. These activities helped facilitate nonverbal communication. Due to her professional background the children’s nurse was able to offer tasks appropriate for the developmental level of a child. From the play and exploratory behavior in at least two different tasks, the level of cognitive development was estimated in the absence of standardized and culturally appropriate tests. Standardized and culturally appropriate tests were not available.

Trainees took notes of the clinical interviews for case discussions in the classroom. The clinical interviews included assessments aimed at detailed and specific descriptions of the developmental status of the observed children. Trainees took part in the clinical assessments by measuring the weight of the child or checking vital signs to detect relevant medical problems (see Approach to children: basic elements).

Approach to children: basic elements

Take weight and height

Look and listen to child and parent

Give tasks to guarantee success

Elicit strengths

Encourage

Determine developmental age

Give a perspective

3. Case discussions: the cases for such sessions were patients seen and written up by MT, or developed from the clinical interviews. Trainees presented the history of the patient and described the mental status to their classmates. Discussions were stimulated by asking questions like: Which additional information would you like to have? What are the differential diagnoses? What are the possible interventions related to the diagnoses? Participants were able to link theoretical knowledge to clinical work during these group discussions. An assessment of strengths and needs of the patient were routinely included for each case.

4. Community orientation: students learned about the importance of the community such as family and school for interventions of child mental health problems. To enhance this experience, visits to community programs were added. In 2012, the course participants visited a school and a well-structured rehabilitation program for street children in Jimma City run by a local branch of the organization “facilitators for change”. In 2013, contact was made with one of the public schools by local faculty (MA) with a plan to include school collaboration in the course. The following case example demonstrates how students were involved in community activities.

A 12 year old boy suffering from seizures explained that he has been rejected at school by peers and teachers, and that he was at risk of dropping out of school. He appeared depressed and tearful during the examination. To support the patient, one mental health professional and one second year postgraduate mental health student went to the school to discuss the patients issue with the school director and teachers.

5. Teaching skills: each of the trainees had to prepare a short presentation on a child mental health condition relevant in Ethiopian health care. The topics were selected jointly by MT and RF (see Child psychiatry course agenda section). Students were instructed to use the course textbook and other scientific literature.

Trainees were advised to focus on a single aspect of a topic and to rely on one page notes to keep within the time frame of the presentation. The presentations were recorded on videotape. After watching the videotape, the presenters were asked first what they liked about their presentation. Trainees learned to begin with giving feedback on the positive aspects of the presentation and to avoid negative criticism. Constructive feedback was given then to the presenter by other trainees and teacher to strengthen teaching skills.

The main contents of these presentations were written on the blackboard. Students learned to consider the expectations and the knowledge base of the audience. Supervision aimed at building links from theoretical knowledge to clinical practice. The teaching exercises took approximately one third of the whole teaching time.

#### Child psychiatry clinical internship

An existing agreement between Jimma University and AAU made it possible for the students do their child psychiatry clinical internship in Addis Ababa for a period of four weeks. A child psychiatrist supervised the trainees at the outpatient clinic of Yekatit 12 Hospital, Addis Ababa. Trainees had to conduct clinical assessments and prepare treatment plans for child and adolescent patients. The cases were presented to their supervisors usually on the same day.

### Outcome of the program

#### Assessment of trainees

For the first year child psychiatry course, the methods of assessment were both formal and informal. Trainees had to assess the mental state of a child at the end of the first week, and develop a plan for management from a short video at the end of the second week. The final test was to develop a five minute presentation on one aspect of intellectual disability, thus combining clinical and didactic aspects. The trainees’ observations were good and the intervention concepts were detailed and realistic. Feedback was given individually and to the group on strengths and areas for further development.

Grades were assigned based on two written tests and on progressive assessment of contributions in group discussions as well as performance on their short presentations. All 25 trainees of the first three successive years passed their child psychiatry course examinations with overall performances ranging from ‘good’ to ‘excellent’. All presentations made by the trainees were systematic in content and presented appropriately.

For the second year child psychiatry clinical internship, students were assessed progressively using a structured format adapted from a clinical assessment tool developed for first year residents in internal medicine at Jimma University. The tool consisted of items on clinical knowledge and skills, ability to develop a plan of management, ability to work with a clinical team, exhibiting respect to patients and their families, proper time management and ethical clinical practice. The students had to score a minimum of 70% to pass. All of the 16 students in the first and second year of the program successfully completed their child psychiatry internship with assessments of ‘good’ to ‘excellent’ by their respective supervisors. The third group of students have not done their child psychiatry clinical internship yet.

#### Assessment of the child psychiatry course by trainees

The classroom teaching activities were assessed by trainees formally by means of a structured feedback sheet and informally through group discussion with the faculty at the end of each week. The evaluation sheet was adapted continuously in order to evaluate each element of the course. Table 1 shows the results of the final evaluation of all three courses. Overall the teaching of child and adolescent psychiatry was rated positive.

**Table 1:** Evaluation of the child and adolescent psychiatry course by trainees

**Year**	**2010**	**2012**	**2013**
**Number of participants**	**5**	**10**	**9**
Make a judgment on the following statements.…			
Scale: 1 = absolutely - 5= not at all	Mean	Mean	Mean
My expectations regarding the course were fulfilled.	1	-	-
The course was worthwhile.	1	-	-
The exchange has widened my knowledge and skills	1	1.2	1.4
I will be able to apply contents of the course in my later professional life	1	1	1.1
In my opinion the course was.…			
Scale: 1 = very good – 5 very poor	Mean	Mean	Mean
Duration	2.4	1.5	1.5
Lectures	-	1.5	1.6
Seminars	-	1.3	1.6
Own presentations	-	1.4	1.3
Video examples	1	1.1	1.4
Activities of patients (painting, etc.)	-	-	1.4
Discussion on patients	-	1.2	1.6
The practical use of proposed solutions	-	1.3	1.4
Working atmosphere	1.2	1.6	1.4
The presenter	1.2	1.6	1.8

The case discussions on video and patient material, and the interactive style of teaching were highly appreciated by trainees. Trainees suggested that a short systematic overview including DSM diagnoses be added at the end of the case discussions. The duration of the course was considered to be too short. Some unexpected feedback by the trainees was that they learned how to better manage their time.

#### Child psychiatry course revision

After the first group of trainees completed the child psychiatry course in 2010, revisions were made based on feedback and staff inputs. In February 2012, the second course was held with modification to increase the time to discuss patient management. Classroom teaching time also was decreased to have more time for clinical activities. In February 2013, the third course was extended to three weeks and modified further to emphasize decision making and problem management. Additionally, the lecture time was reduced. Compared to previous courses, there was enough time to discuss the management of patients in depth during the third year course.

Over the three years, both guest lecturers increased their understanding of the culture and customs of the country as well as the environment at the psychiatric clinic. They learned about traditional healing methods such as giving holy water or treatment with herbs, and customs such as the use of khat. Translation to and from the local languages, Amharic and Afaan Oromo were still needed. They were better able in adapting their teaching to the local situation and to link clinical and teaching activities.

As a result of the modification to the course, the education and learning of the trainees appeared to improve. For example, during the first course it was difficult for the students to assess and describe the cognitive level of children. In subsequent years, having the trainees learn about various interactive assessment methods before working with the children facilitated the students’ ability to evaluate youth accurately.

#### Follow-up of graduates

Graduates who completed the first and second course are working in different public institutions in Ethiopia. The trainees of the third course were still in their second year of their MSC training. Follow-up feedback was obtained from program graduates. Out of the sixteen graduates, four are working in the northern (Tigray region), two in the northeast (Amhara region), and four in the eastern (Amhara and Harari regions) parts of Ethiopia. Moreover, five were employed as faculty at the Department of Psychiatry, Jimma University (southwest, Oromiya region). Although all five have additional clinical responsibilities and opportunity to provide clinical care for child psychiatric patients, one of them (MA) has taken the main responsibility to establish an outpatient clinic for children and adolescents. In addition, he is doing a research project on childhood behavioral/emotional problems and their relation to academic performance, as well as how the primary school teachers perceive child mental health problems. MA has also been involved in co-teaching the child psychiatry course with RF and CGF.

Six graduates are working as faculty in different regions at governmental universities teaching undergraduate nurses and health officers primarily with limited clinical activities. Two graduates are practicing clinical psychiatry within regional/zonal/hospitals in different areas of the country. Two are working as faculty of universities affiliated with referral hospitals and see patients. Another graduate is working at a student health clinic of a university providing psychiatric care for the university students. Only two of the above mentioned work places employed previous psychiatric service providers. The participants of the third course have been seeing child patients since completing the child course, and express confidence in caring for child patients. Other feedback included suggestions such as reducing the course content and increasing specific subject content for example epilepsy and enuresis.

## Discussion

Training in child psychiatry was successfully developed and implemented within the curriculum for graduate training in mental health for non-physician clinicians. We found the child psychiatry sections of mhGAP-IG to be very helpful in training non-physician mental health specialists in Ethiopian setting. The early results indicated very good outcomes in clinical knowledge and skills, and satisfactory improvement in human resource for child mental health in the health care system. Furthermore, it presented important resource for the implementation of the training of primary care workers in the scale up of mhGAP at a national level.

Several factors have contributed to the successful development and implementation of the training. The involvement and support of various stakeholders, the commitment of various collaborators; and the financial and material support of Jimma University and CIH-LMU were crucial in realizing the program in the setting of Department of Psychiatry, Jimma University.

The consultative meeting at the early stages of program development enabled better understanding of the existing child mental health needs and mobilized support from potential collaborators. The fact that FMOHE was in support of this project meant that the regional health bureaus were able to recruit and sponsor potential candidates for training, and that the Jimma University could fund the program implementation.

Jimma University’s strong financial and material support made the development of the course possible along with support from CIH-LMU and AAU. Having available and accessible internet and computer service at Jimma University made it possible to communicate with experts elsewhere in the world during the process of curriculum development. The latter was significant in two ways: first, the child psychiatry training part of the draft curriculum could further be developed by experts in collaborating institutions; second, communication during curriculum development with collaborators enabled ‘smooth’ implementation of the training. Moreover, availability of internet service facilitated access to electronic resources for the trainees and the faculty.

The launch of CIH at LMU which paralleled the launch of the MSC program at Jimma University facilitated having visiting professors in Jimma teach the child psychiatry course. In addition, AAU had started a new child and adolescent psychiatric service at Yekatit 12 hospital in Addis Ababa. The latter created an excellent opportunity for the trainees to do a clinical internship in child psychiatry under the supervision of an Ethiopian child psychiatry specialist. Successful institutional collaboration in creating the psychiatry residency program at AAU has been described previously [21].

The use of WHO mhGAP-IG as the main training material for the child psychiatry course has enabled the successful delivery of skill focused training. In addition, as the national mental health strategy of Ethiopia is promoting the scaling up of psychiatric services through integration of mental health into primary care [7], the graduates will be excellent resources for the health care system. They could potentially train, and provide supportive supervision to primary care workers within their respective catchment areas. A recent study has reported that one of the barriers to integrating mental health care into the primary care was a lack of supportive supervision and the inadequate mental health knowledge of general health workers [Abera M, Tesfaye M, Belachew T, Hanlon C: Perceived Challenges and Opportunities by Primary Healthcare Workers about Integrating Mental Health care into Primary Health care; Jimma zone, South Western Ethiopia, submitted].

The program succeeded in training child psychiatric clinical knowledge and skills within a relatively short period of time through emphasis on clinical skills training using practical teaching strategies and to a lesser extent didactic teaching. A Canadian child psychiatrist who taught a course in child psychiatry at Addis Ababa University in 2008 had to adapt the content to the relevant Ethiopian context although teaching approaches did not have to be modified [22]. The successful completion of all trainees in their child psychiatry clinical internship with good results several months after the child psychiatry course was conducted suggests that the clinical skills were maintained. In contrast to the three month clerkship recommended in high income settings our internship is shortened by two months [23]. Nonetheless, our results demonstrate positive changes in attitude among the trainees [24]. Future studies evaluating its effects on the child psychiatric practice of graduates will provide valuable information on designing cost effective models of child psychiatric training for low income settings.

Both the trainees’ successful completion of the courses and their feedback indicate positive outcome in terms of acquired knowledge and skills as well as trainees satisfaction with their training in child psychiatry. However, the impact of the training in the health system should be cautiously interpreted since it is too early to come to any conclusions. It is encouraging to see that many of the graduates have been actively practicing. The fact that six graduates working in four different universities have limited opportunities to see patients clinically is a potential threat to the efficient utilization of their skills. Graduates who are involved in teaching nurses and health officers at the universities are presented with opportunities to convey child psychiatric skills and knowledge. These graduates have possibly benefitted from the ‘teaching skills’ training element of the child psychiatry course.

### Future directions

Local faculty development in conjunction with the strengthening of child psychiatric services have been the priorities in this undertaking [25]. The only graduate who had committed to child psychiatry has expressed a need for additional staff for sustainable teaching, providing clinical service and establishing inter-disciplinary collaboration with other services. The long-term goal is for the child psychiatry course and clinical internship to be fully delivered within the Jimma University system by local faculty and staff. Achieving that goal needs time, persistence and commitment by all relevant stakeholders.

## Conclusion

Child psychiatry training for non-physician mental health specialist trainees was developed and successfully implemented through collaboration among several universities. The model of institutional collaboration in providing training to improve human resource for mental health in the context of limited resources provides a useful guide for other low income countries where there is scarcity of psychiatrists. Building local capacity, particularly in the area of development of local faculty should be the next step to ensure the sustainability of these training programs in these settings.

## Competing interests

The authors declare no competing interests.

## Authors’ contributions

MT was involved in conception, design and write up of the manuscript. MA was involved in the design, data collection and write up of the manuscript. CGF was involved in the design and reviewed the manuscript. RF was involved in the conception, design, data analysis and write up of the manuscript. All authors have read and approved the final manuscript.
